# Effect of Chronic Resistance Training on Circulating Irisin: Systematic Review and Meta-Analysis of Randomized Controlled Trials

**DOI:** 10.3390/ijerph18052476

**Published:** 2021-03-03

**Authors:** Pedro L. Cosio, Manuel Crespo-Posadas, Álvaro Velarde-Sotres, Mireia Pelaez

**Affiliations:** 1 Faculty of Health Sciences, Universidad Europea del Atlántico, 39011 Santander, Spain; manuel.crespo@uneatlantico.es (M.C.-P.); alvaro.velarde@uneatlantico.es (Á.V.-S.); mireia.pelaez@uneatlantico.es (M.P.); 2 National Institute of Physical Education and Sport of Catalonia (INEFC), University of Barcelona, 08038 Barcelona, Spain

**Keywords:** exercise therapy, metabolic diseases, peptide hormones, immunoassay, muscle fibers

## Abstract

Irisin seems to play an important role in several chronic diseases, however, the interactions between chronic training and irisin are still unclear. The purpose of this systematic review and meta-analysis was to examine the effect of chronic resistance training on circulating irisin in adults. Literature search was conducted in PubMed, Web of Science and EBSCOhost (Academic Search Complete) until December 2020. Randomized controlled trials researching irisin levels after a resistance training program for at least 8 weeks among an adult population were eligible. Other inclusion criteria comprised recruiting a control group and reporting circulating irisin through ELISA kits. Cohen’s d effect size and subgroup analyses (95% confidence level) were calculated using a random effects analysis model. Data of the seven included studies comprising 282 individuals showed an increasing and non-significant tendency after a resistance training program (*d* = 0.58, 95% CI: −0.25 to 1.40, *p* = 0.17). Subgroup analyses showed significant increases for the older adults group (*p* < 0.001) and when training is demanding and progressive in terms of intensity (*p* = 0.03). Data suggest that resistance training programs seem to increase circulating irisin, especially in older adults and in demanding and progressive training programs. However, more studies should be conducted using robust measurement methods, such as mass spectrometry, to better understand the interaction between chronic resistance exercise and irisin.

## 1. Introduction

Myokines such as IL-15, IL-6, IL-8, brain-derived neurotrophic factor, irisin, etc. are secreted by skeletal muscle cells and, as other cytokines, are designed to regulate inflammation and immune responses [1,2]. There is a complex interaction among cytokines and a comprehensive model remains to be elucidated. Irisin is a recently identified peptide hormone derived from the extracellular domain of the fibronectin domain-containing 5 protein (FNDC5) [3,4]. Since its discovery by Boström et al. [5], irisin has been largely studied regarding its potential therapeutic aspects. It takes part in the transformation of white fat tissue into brite/beige fat cells via uncoupling protein -1 (UCP-1) [6]. The resultant beige adipocytes are rich in mitochondrial UPC-1 and specialize in heat production and energy expenditure [7]. It has also been shown to control mitochondrial biogenesis and oxidative metabolism in many cell types [5]. Irisin increases glycerol release and reduces lipid accumulation in adipocytes and, additionally, increases GLUT-4 expression in beige fat cells, increasing their glucose uptake capacity [8]. For these reasons, irisin has been suggested as a therapeutic hormone for obesity and other metabolic conditions [9].

Literature on anti-inflammatory, anti-oxidative and anti-apoptotic effects of irisin in various pathological conditions such as obesity, type 2 diabetes, non-alcoholic fatty liver disease, chronic liver disease, osteoporosis, hypertension, atherosclerosis, Alzheimer, and cancer, shows promising results [10,11,12,13,14,15,16,17,18]. Physical inactivity and increased abdominal adiposity are associated with persistent systemic low-grade inflammation [19]. On the other hand, exercise has been considered as an anti-inflammatory therapy due to the fact that it releases anti-inflammatory myokines into circulation [20,21]. Therefore, a better understanding of irisin and its secretion may have a notable impact on public health.

The frequency, intensity, time, and type (FITT) of the exercise that modulates irisin secretion have been previously studied. Qiu et al., 2015 conducted a meta-analysis intended to analyze the chronic effect of physical exercise on circulating irisin [22]. Resistance training resulted in decreased circulating irisin in randomized controlled trials (RCTs) and non-significant changes in non-randomized trials. However, their analysis included only two RCTs, and thus could not consider possible moderator variables.

Considering that the main source of FNDC5/irisin is muscle mass [5,23], which accounts for approximately 72% of its secretion [5], and that previous articles have shown a transient increase in circulating irisin in the first two hours after a single resistance training session in healthy adults [24,25], we suggest that the cumulative acute effects produced by each training session may be sufficient to lead into temporary adaptations over time. Due to the complexity of resistance training variables, meta-analyzing articles investigating resistance training appears to be problematic [26]. However, this issue can be addressed using strategies to deal with heterogeneity such as subgroup analysis [26]. New RCTs have been published since the previous meta-analysis was conducted [22] and, consequently, moderator variables can be analyzed to address the natural heterogeneity of resistance training programs. Thus, a meta-analytical approach to the chronic effect of resistance training on circulating irisin could extend the scientific knowledge on the topic for a clear comprehension of the interaction between exercise and irisin. Therefore, the purpose of this systematic review and meta-analysis is to examine the chronic effect of resistance training on circulating irisin in an adult population.

## 2. Materials and Methods

This Systematic Review and meta-analysis was developed in accordance with the Preferred Reporting Items for Systematic Reviews and Meta-Analysis protocols (PRISMA-P) statements 2015 [27] and has been registered with PROSPERO (CRD42020202551). This ensures that both the systematic review and the meta-analysis follow the pre-established steps and contain the specific sections to ensure its reproducibility (see Table S1).

### 2.1. Search Strategy

The search was conducted using MEDLINE (PubMed), Web of Science and EBSCO (Academic Search Complete) databases from January 2012 to December 2020. The search strategy included the words “irisin” and “FNDC5” combined employing the boolean command “AND” with the words “training”, “resistance training” or “exercise” (see Figure S1). References from other articles or major reviews were also verified to ensure the presence of all relevant RCTs.

### 2.2. Inclusion and Exclusion Criteria

Articles eligible for inclusion in the systematic review had to meet the following inclusion criteria: (1) be RCT; (2) be conducted in population without major diseases (trained or untrained) over 18 years of age; (3) perform a resistance training intervention of at least 8 weeks of duration; (4) be compared with a control group; and (5) report circulating irisin through immunoassays (ELISA). Research was excluded if it did not meet the inclusion criteria, was written in a language other than English, or was infographic-type, case-study, or other similar forms of scientific dissemination due to limited information.

### 2.3. Study Selection and Data Collection

The selection of studies for inclusion was conducted by two independent reviewers (P.L.C. and A.V.-S.), and in case of disagreement, a third reviewer (M.P.) resolved the disparity. Once the papers that met all the established inclusion criteria had been found, the following data were extracted and noted in a Microsoft Office Excel 2019 spreadsheet: (1) Authors; (2) Year of publication; (3) Characteristics of the participants (age, BMI, gender, other); (4) Characteristics of the resistance training program (e.g., frequency, intensity, duration, number and type of exercise, sets and repetitions, supervision); (5) Adherence to the protocol; (6) ELISA kit used and time to blood collection; (7) General conclusion.

Authors whose papers did not meet the required data for qualitative and quantitative synthesis were contacted by e-mail or ResearchGate and asked about the possibility of sharing the information not included in their published studies.

### 2.4. Assessment of Risk of Bias

The quality of each included article was evaluated by two independents reviewers (P.L.C. and A.V.-S.) using version 2 of the Cochrane tool for risk of bias in RCTs (RoB 2), which is based on a series of risk domains that focus on aspects of trial design, conduct, and reporting [28]. The tool includes five domains that evaluate (1) bias arising from the randomization process; (2) bias due to deviations from the intended interventions; (3) bias due to missing outcome data; (4) bias in measurement of the outcome; and (5) bias in selection of the reported result. Each of these domains is assigned a low risk of bias, some concerns, or high risk of bias assessment. Based on the assessments obtained in each of the five domains, each included study is classified as low risk of bias (low risk of bias for all domains), some concerns (some concerns in at least one domain) or high risk of bias (high risk of bias in at least one domain or some concerns for multiple domains). Considering that the nature of the studies implies an intervention with physical exercise in comparison with a control group, it is impossible for the study participants to be blinded. Therefore, the criterion of participant blinding was not considered. In addition, other confounding variables were considered for the qualitative analysis, such as age, sex, body mass index (BMI), supervision, and adherence to training, since these could influence and explain certain results.

### 2.5. Statistical Analysis

Calculating the effect sizes for each study was based on the standardized mean difference (Cohen’s d) for independent groups. The proposal by Morris [29] was selected, which uses the mean change between pre- and post- irisin concentrations in the experimental group minus the mean change between pre- and post- in the control group, divided by the combined standard deviation at the pre-intervention time. Subsequently, the overall effect size was established, with 95% confidence intervals. For this purpose, a random effects model was run in Review Manager (RevMan) (https://training.cochrane.org/online-learning/core-software-cochrane-reviews/revman/revman-non-cochrane-reviews (accessed on 3 March 2021).), version 5.4.1, The Cochrane Collaboration, 2020, since this analysis technique ponders each study by the inverse of its variance and incorporates heterogeneity among the studies included into the model. The effect size is conventionally translated as large for values of 0.8; moderate for values of 0.5; and small for values of 0.2 [30].

Subgroup analyses were performed in order to assess the potential effects of moderator variables. A priori selected moderator variables of age; gender; body weight (change), BMI (change); % body fat (change); intervention duration; intensity; progression in intensity; supervised training sessions; article risk of bias; and journal rank were included in the subgroup analysis. First, age was transformed into a categorical variable formed by a group of adults (up to 60 years) and a group of older adults (from 60 years). In the case of body weight, BMI and % body fat, they were divided based on their change over the period of training (increase/same/decrease). Intervention duration (≤12 weeks/>12 weeks) were divided using a median split, while supervised training sessions (≤80%/>80%) were divided using the minimum percentage of assistance to achieve benefits. Intensity was classically divided (≤60%/61–85%/>85%), and finally, gender (male/female), progression in intensity (yes/no) article risk of bias (low risk/some concerns/high risk) and journal ranking (Q1/Q2/Q3/Q4) were divided into their natural categories.

Heterogeneity among the studies was evaluated using the Cochrane Q test, considering *p* < 0.1 as statistically significant, and the I^2^ statistic, which describes the percentage of total variation among the studies that is due to heterogeneity. In this way, the values of 25%, 50% and 75% were considered as low, medium, and high heterogeneity, respectively [31]. A sensitivity analysis was performed to assess the robustness of the results eliminating each study one by one. The presence of scientific bias was evaluated through the Egger test [32] and the Begg test [33], considering *p* < 0.05 a statistically significant bias in both cases. If publication bias were detected, the non-parametric Trim and Fill analysis [34] would be applied, with the aim of estimating the impact of publication bias on the results.

## 3. Results

### 3.1. Study Selection

**Figure 1:**
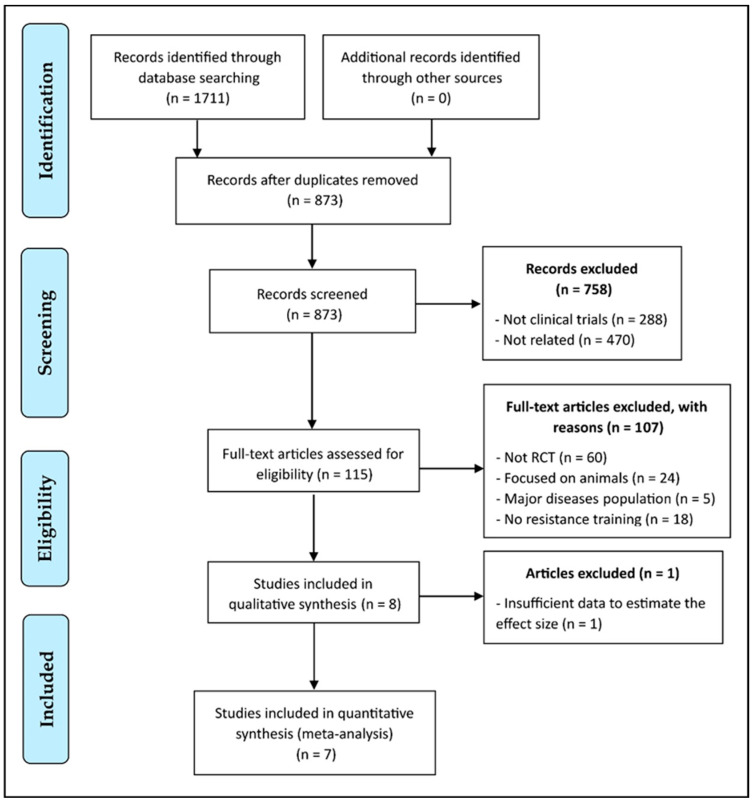
Flowchart of study selection according to the PRISMA Guidelines. RCT: Randomized Controlled Trial.

A total of 1711 citations were found after applying the search criteria in the corresponding databases. After removing duplicates with the Mendeley Desktop software tool, 873 scientific papers were identified. Of these, 758 were discarded after reading their title and abstract, either because they were not relevant, because they were review articles, or because they were written in a language other than English. The complete texts of the remaining 115 articles were evaluated in more detail to see if they fully met the inclusion criteria. A total of 107 articles did not meet the complete established inclusion criteria, either because of their design, population, or proposed intervention. No additional studies were identified after verifying the references of the articles included or through other additional sources. Therefore, eight articles [35,36,37,38,39,40,41,42] met the inclusion criteria and were incorporated into the qualitative analysis [40]. Nevertheless, one article ultimately did not present enough data to estimate the effect size [40], so the quantitative synthesis included seven articles [35,36,37,38,39,41,42]. The literature search and selection of studies are presented in Figure 1.

### 3.2. Characteristics of the Included RCTs

A total of 355 participants were involved in the 8 RCTs. Of these, two analyzed both sexes (25%), another four analyzed only women (50%), and two analyzed only men (25%). The participants in all articles were adults over 18 years of age, without major diseases or pathologies, physically inactive, and randomly assigned to a control or experimental group. The main differences between the resistance training protocols conducted in the included articles can be seen in Table 1.

Although all the interventions proposed similar frequency, training volumes, super-vised session percentages and multi-articular exercises, the intensity, the progression in that intensity and the duration of the training schedules differed between the interventions. The intensity of the training was in a range between 55% [39] and 85% [38,41] of 1RM, and five of the eight articles considered the progression in training intensity [37,38,40,41,42]. Considering the type of exercises performed, most of the trainings were executed using machine-based exercises [35,36,38,40,41,42], while the program of Kim et al., 2015 [37] was executed using elastic bands; and that of Ghanbari-Niaki et al. [39] was performed in circuit mode, and therefore, the intensity could be affected. Finally, the duration of the resistance training program oscillated from 8 weeks [39,42] to 26 weeks [35].

### 3.3. Assessment of Risk of Bias

After assessing the risk of bias, 100% of the included studies showed low risk of bias in the domains of “randomization process”, “deviations from the intended interventions”, “missing outcome data” and “selection of the reported result”. However, the domain related to measuring the outcome presented some concerns in five of them [36,37,38,39,40]. This is because one of the parameters that this domain evaluates is the evaluators data blindness. In these studies [36,37,38,39,40], the outcome assessors were aware of the intervention received by study participants. However, it is unlikely that assessment of the outcome was influenced by the knowledge of the intervention received. In the overall judgment, three studies presented low risk of bias [35,41,42], while the rest of the studies [36,37,38,39,40] present some concerns regarding risk of bias assessment (Table 2).

### 3.4. Chronic Effect of Resistance Training on Circulating Irisin

The qualitative analysis of the studies included in the systematic review consisted of eight studies [35,36,37,38,39,40,41,42] as detailed in Table 1. In general, three of them did not confirm a greater increase in circulating irisin after a resistance training program compared to the control group [35,36,42], another found non-significant increases (*p* = 0.079) in the resistance training group [40], and the remaining four showed significant increases in circulating irisin in the resistance training groups [37,38,39,41].

The interventions that did show improvements in the control group compared to the resistance training group [35,36] had a long intervention duration (approximately 6 months). However, the intensity of their exercises was significantly low. Furthermore, their training protocols were similar, except for the progression of the loads, since Hecksteden et al. [35] did not progress and Scharhag-Rosenberger et al. [36] only progressed in the volume of training. It is important to note that the participants of these studies who performed the resistance training program increased circulating irisin levels, but the control groups experienced larger increases.

The interventions that did find greater increases in irisin levels in the resistance training group coincided in a shorter duration of the intervention (8–12 weeks). Nevertheless, they established training progressions. Kim et al. [37] and Amanat et al. [41] showed progression in volume and training intensity, and the program of Zhao et al. [38] gradually increased training intensity. On the other hand, all the studies conducted in the older population found increments in circulating irisin in favor of the resistance training group. In addition, of the studies conducted in the older adults, Kim et al. [37] included only women, while Zhao et al. [38] included only men. Lastly, all participants in the included studies presented similar BMI and were physically inactive. Therefore, no differences were found in the results of the qualitative synthesis according to the level of previous physical activity, BMI or ELISA kit used.

#### 3.4.1. Meta-Analysis

**Figure 2:**
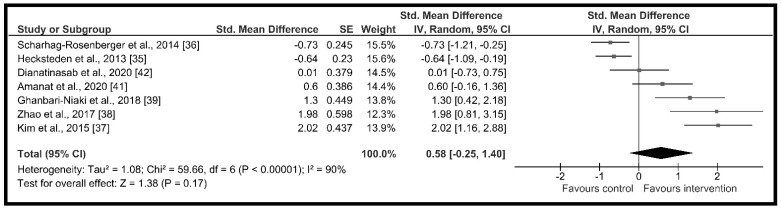
Forest plot of RCTs investigating the chronic effect of resistance training programs on circulating irisin [35,36,37,38,39,41,42]. Squares represent the standardized mean difference for each study and diamond represents the overall estimated effect across the studies. CI: confidence interval, IV: inverse-variance.

A comparison between seven studies [35,36,37,38,39,41,42] was used to evaluate the effect of resistance training on irisin serum level concentrations. High heterogeneity was obtained among the studies included in the quantitative synthesis (Q = 59.66, *p* < 0.01; I^2^ = 90%), by which the overall effect size was calculated using the random effects method. The results of the meta-analysis indicated a moderate and non-significant effect of resistance training programs in increasing circulating irisin, as indicated by the overall effect size in Figure 2 (*d* = 0.58, 95% CI: −0.25 to 1.40, *p* = 0.17).

#### 3.4.2. Effect of Moderator Variables

**Figure 3:**
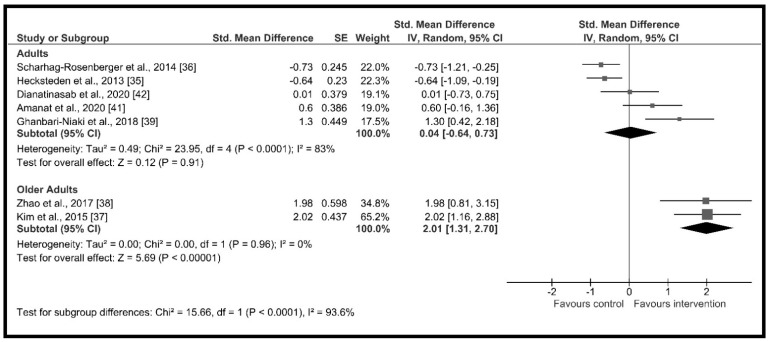
Forest plot of RCTs investigating the chronic effect of resistance training programs on circulating irisin according to age subgroup [35,36,37,38,39,41,42]. Squares represent the standardized mean difference for each study and diamonds represent the subgroup estimated effect across the studies. CI: confidence interval, IV: inverse-variance.

**Figure 4:**
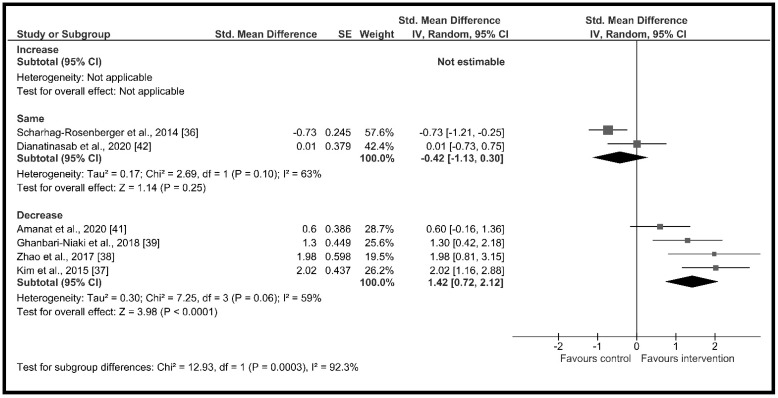
Forest plot of RCTs investigating the chronic effect of resistance training programs on circulating irisin according to change in body fat (%) subgroup [36,37,38,39,41,42]. Squares represent the standardized mean difference for each study and diamonds represent the subgroup estimated effect across the studies. CI: confidence interval, IV: inverse-variance.

**Figure 5:**
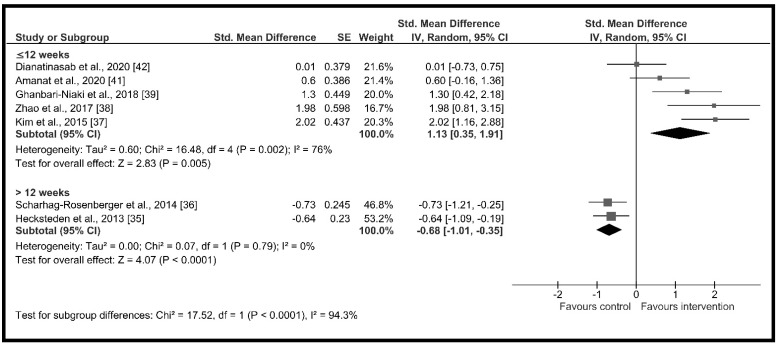
Forest plot of RCTs investigating the chronic effect of resistance training programs on circulating irisin according to intervention duration subgroup [35,36,37,38,39,41,42]. Squares represent the standardized mean difference for each study and diamonds represent the subgroup estimated effect across the studies. CI: confidence interval, IV: inverse-variance.

**Figure 6:**
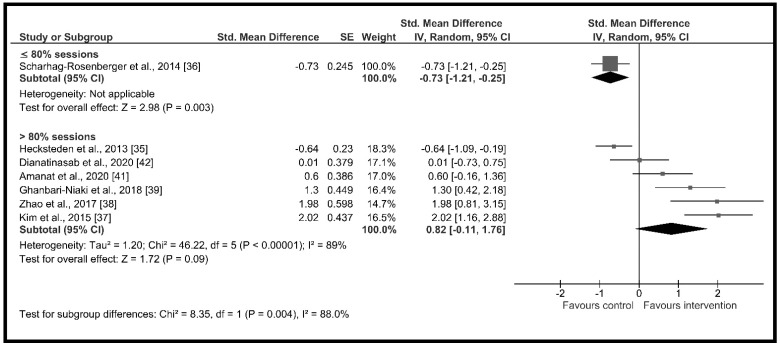
Forest plot of RCTs investigating the chronic effect of resistance training programs on circulating irisin according to supervised sessions (%) subgroup [35,36,37,38,39,41,42]. Squares represent the standardized mean difference for each study and diamonds represent the subgroup estimated effect across the studies. CI: confidence interval, IV: inverse-variance.

The high heterogeneity (I^2^ = 90%) suggested the presence of potential moderators. Therefore, subgroup analysis showed significant differences across groups according to (a) age (*χ*^2^ = 15.66, *p* < 0.001) with a high and significant increase in circulating irisin in the older adults group (*d* = 2.01, 95% CI: 1.31 to 2.70, *p* < 0.001) and a non-significant increase in adults group (*d* = 0.04, 95% CI: −0.64 to 0.73, *p* = 0.36) (Figure 3); (b) change in % body fat (*χ*^2^ = 12.93, *p* < 0.001) with non-significant changes in circulating irisin when the % body fat did not change during the intervention period (*d* = −0.42, 95% CI: −1.13 to 0.30, *p* = 0.25) and a high and significant increment when the % body fat decreased throughout the intervention period (*d* = 1.42, 95% CI: 0.72 to 2.12, *p* < 0.001) (Figure 4); (c) intervention duration (*χ*^2^ = 17.52, *p* < 0.001), with a high and significant increase in circulating irisin when the duration of the intervention was less or equal to 12 weeks (*d* = 1.13, 95% CI: 0.35 to 1.91, *p* = 0.005) and moderate and significant decreases when it lasted more than 4 months (*d* = −0.68, 95% CI: −1.01 to −0.35, *p* < 0.001) (Figure 5); and (d) supervised sessions (*χ*^2^ = 8.35, *p* = 0.004) with a moderate and significant decrease in circulating irisin when less than 80% of the sessions were supervised (*d* = −0.73, 95% CI: −1.21 to −0.25, *p* = 0.004) and high but non-significant increases when more than 80% of the sessions were supervised (*d* = 0.82, 95% CI: −0.11 to 1.76, *p* = 0.09) (Figure 6).

Other relevant results are that both higher intensity training programs (61 to 85% 1RM), and those that plan a progressive increase in intensity, result in significant increases in irisin (*d* = 1.10, 95% CI: 0.11 to 2.09, *p* = 0.03). Conversely, no significant differences between groups were found according to gender (*χ*^2^ = 1.90, *p* = 0.17); change in body weight (*χ*^2^ = 0.83, *p* = 0.36); change in BMI (*χ*^2^ = 0.01, *p* = 0.93); article risk of bias (*χ*^2^ = 1.72, *p* = 0.19); and journal rank (*χ*^2^ = 4.82, *p* = 0.09). A summary of the effect of moderator variables can be viewed in Table 3.

#### 3.4.3. Sensitivity Analysis and Publication Bias

By removing each study one by one from the meta-analysis, the re-pooled overall effect size showed that none of the studies substantially modify the results, neither in their magnitude, nor in their significance nor in their heterogeneity. Publication bias was detected after performing both the Begg test (Begg = 15.00, *p* = 0.04) and Egger test (Egger = 8.73, *p* < 0.001), by which the Trim and Fill procedure was applied to correct the impact of publication bias. This analysis imputed one study on the left side of the 0 line, so the correction of the overall effect size is *d* = 0.34, 95% CI: −0.45 to 1.13, *p* > 0.05. This correction does not substantially modify the result, neither their magnitude, nor their significance nor their heterogeneity.

## 4. Discussion

### 4.1. Main Findings and Interpretation

To our knowledge, this is the first systematic review and meta-analysis focused on the chronic effect of resistance training on circulating irisin and its possible moderator variables. The quantitative analyses showed a non-significant positive trend of serum irisin after a resistance training program. However, subgroup analyses revealed (a) a very high and significant increase of circulating irisin in the older adults subgroup; (b) a high and significant increase when the % body fat decreased across the intervention period; (c) a high and significant increase in circulating irisin when the duration of the intervention was less or equal to 12 weeks and moderate and significant decreases when it lasted more than 4 months; and (d) a moderate and significant decrease in circulating irisin when less than 80% of the sessions were supervised.

The results of our analysis are in contrast with other authors. The meta-analysis published by Qiu et al. [22] showed that chronic exercise training had a moderate and significant effect in decreasing circulating irisin in adults in the RCTs that they analyzed, although non-significant differences were found in the non-RCTs. However, their analysis only includes two articles related to the chronic effect of resistance training. Furthermore, since 2015, new RCTs meeting the inclusion criteria have been published adding new relevant data which had modified their previous results. Another review indicated that chronic exercise appears not to affect circulating irisin [43]. Nevertheless, their analysis included protocols consisting of either aerobic or resistance training, so conclusions should be taken with caution.

Regarding to our analysis, the articles that did find significant decreases in circulating irisin present some characteristics in common [35,36]. First, its duration was very long (6 months approx.). However, the intensity of the training sessions was remarkably low, and the progression in the intensity of the training was not considered. It is important to note that the participants of these studies who performed the resistance training program increased circulating irisin levels, but the control groups experienced a surprising and disproportionate increase in circulating irisin. In the case of articles that found significant increases in circulating irisin, the duration of the intervention was lower (2–3 months ap-prox.), but training intensity was more demanding and increased throughout the pro-gram.

Results from other studies not included in our analyses showed both increases [44] and no changes [45] in circulating irisin after 8 weeks of resistance training. Specifically, a pilot study by Kim et al. [44] not included in this review since it did not completely meet the criteria for randomization, reinforces the results obtained in our analysis. They found an increase in circulating irisin in the resistance training group compared to the aerobic training group and the control group (*p* < 0.001). It should be noted that the intensity of the training was demanding and progressive with the capacities of the participants. These different results between studies show that the scientific literature is still too divergent, probably due to a possible multifactorial response of irisin. Actually, irisin existence have been widely questioned [46] ascribing its existence to a low specificity of the detection methods by antibodies [47]. Nevertheless, it has been demonstrated by tandem mass spectrometry that human irisin exists, circulates, is regulated by exercise, and that it has a remarkably similar or identical structure to mice protein [48].

A possible increase in irisin levels after a resistance training program as a result of increased muscle tissue makes some sense due to authors such as Huh et al. [23] or Chang et al. [49] highlight fat-free mass as one of the main predictors of circulating irisin. This idea is consistent with the results of our subgroup analysis, which indicated significant increases in circulating irisin in the participants who decreased their body fat percentage through the resistance training program (*p* < 0.001). In addition, of the four studies that found no increases in circulating irisin, two did not obtain improvements in body composition [36,42] and the other two did not analyze it [35,40]. Therefore, the significant in-crease in circulating irisin in older adults after a resistance training program (*p* < 0.001) is not surprising, in view of the fact that every year, between 1% and 2% of muscle mass be-gins to be lost since from the age of 50 [50]. Although circulating irisin appears to be high-er in women than in men after adjusting for fat-free mass [51,52], subgroup analysis showed no differences between gender in the response of circulating irisin after a resistance training program (*p* = 0.17).

Surprisingly, a long duration of the resistance training program could be negative for circulating irisin (*p* < 0.001). However, according to our results, higher intensity (60 to 85% 1RM) and progressive resistance training programs result in significant increases in circulating irisin (*p* = 0.03). On the other hand, resistance training programs ranged from 10 to 52 weeks at an average intensity of 75% 1RM similarly increase lean body mass in older adults [53]. Therefore, the decreased circulating irisin showed in lengthier resistance training programs in our analysis should be considered with caution because only two articles contribute to these results and they are characterized by a non-progressive lower intensity trainings. Moreover, it has to be taken into account that one did not analyze changes in body composition [35] and the other did not find significant changes [36]. Consequently, potential increases in irisin concentrations may not remain constant if the principle of load progression during a medium/long term training period is not respected. One possible explanation for why shorter training protocols [37,38,39,41] increased circulating irisin could be that myokine secretion, as a result of muscle contraction during physical exercise, followed a cascade profile. In this way, certain myokines, such as irisin, would be secreted initially while others would delay their increase over time. Another explanation may be provided by Hecksteden et al. [35], who considered the possibility that irisin is a molecule with a short life expectancy as a consequence of its high degradation rate.

Both training supervision and adherence must also be considered. There are several studies and meta-analyses that show how supervised training programs are more effective than home-based protocols in different types of populations [54,55,56] and that strength gains are greater under higher supervision ratios [57]. There are authors who also highlight the need for an 80% minimum adherence to ensure optimal gains in strength [58], confirming the importance of direct supervision during resistance training.

On the other hand, all participants in the sample were physically inactive, so the results of this research would only be extrapolated to that population. Considering that, according to Handschin and Spiegelman [59], the expression of the PGC1-α protein seems to be reduced in sedentary subjects, and that irisin is a PGC1-α-dependent myokine, it might be logical to think that trained people will present higher circulating irisin. Therefore, a prospective study comparing the chronic effect of resistance training in trained and untrained people may be interesting. Another variable that seems to affect irisin levels is the time of day. Anastasilakis et al. [51] found, without explanation, a pattern in which the lowest irisin levels were recorded at 06:00 h, with peak concentrations at 21:00 h. Finally, irisin measurements of the included articles were conducted predominantly using the ELISA kits commercialized by Phoenix Pharmaceuticals (Burlingame, CA, USA). The suitability of these ELISA kits for measuring circulating irisin appears to be controversial, since Albrecht et al. [47] found potential cross-reactivity of irisin antibodies against other non-irisin proteins in serum or plasma, and Montes-Nieto et al. [60] reported weak correlations between two different ELISA kits. Despite the fact that the antibodies used by the Phoenix Pharmaceuticals ELISA kits have been validated using mass spectrometry [61,62], Albrecht et al. [63] have again reported in 2020 low reliability of the antibodies used in ELISA kits. Therefore, all results from ELISA kits measurements should be cautiously interpreted until the reliability of irisin antibodies in ELISAs can be proven.

The results of the present systematic review and meta-analysis suggest that older adults showed greater increases of circulating irisin than adults of other ages after a resistance training program. Moreover, these increases in circulating irisin seem to be affected by some training characteristics like intensity, progression in intensity or supervision ratio. Therefore, qualified physical exercise professionals can use resistance training as an anti-aging tool, provided it is systematized and supervised.

### 4.2. Strengths and Limitations

The strengths of this review are: (a) the use of RCTs; (b) the focus in only one type of training (resistance training); and (c) the analysis of the moderator variables on circulating irisin. Likewise, our study has some limitations: (a) the relatively small number of studies; (b) the differences in the resistance training protocols, either in terms of training intensity, progression in intensity and duration in weeks; (c) the participants included were physically inactive, so the results cannot be extrapolated to trained population; and (d) the instrument used to measure circulating irisin in all studies (ELISA kit) is controversial, and therefore the importance of considering it when interpreting the results is highlighted.

### 4.3. Suggestions for Future Research

Given the limitations that our study has faced, and the gaps in scientific literature, the following recommendations for future research may be considered. First, well-design RCTs with more demanding resistance training protocols in terms of intensity and load progression. Second, irisin measurements not only at baseline and post-intervention, but also during the training program, especially in those protocols longer than 8 weeks, explaining in detail the time of day of the measurements. Third, trials comparing untrained and well-trained people to better understand the role of exercise in the dynamics of irisin.

## 5. Conclusions

The findings of this systematic review and meta-analysis suggest a trend for increased irisin after a resistance training program. These increases seem to be accentuated in older adults and when training intensity is demanding and progressive. Given the important limitation in the method of measuring circulating irisin levels, these results are required to be cautiously interpreted and further confirmed by a robust measurement method such as mass spectrometry.

## Figures and Tables

**Table 1:** Overview of studies included in the systematic review and meta-analysis.

Study, Year	Study Group	*N* (m/f)	Age (Years) ^a^	BMI (kg/m^2^) ^a^	D (Weeks)	F (Supervision)	Intervention	I	P	Irisin Measurement (Time Period ^b^)
Hecksteden et al., 2013 [35]	Exp	17/23	48 ± 7.0	24.9 ± 3.4	26	3 (100%)	8 machine-based exercises (2 s × 15 r)	100% 20RM	No	Serum ELISA ^c^ (48 to 168 h)
	Con	13/26	50 ± 7.0	24.5 ± 3.1			Not to change their previous lifestyle			
Scharhag-Rosenberger et al., 2014 [36]	Exp	17/20	47 ± 7.0	25.0 ± 3.4	24	3 (33%)	8 machine-based exercises (2 s × 16/18/20 r in 6-wk cycle)	100% 20RM	Yes (V)	Serum ELISA ^c^ (48 to 168 h)
	Con	12/25	50 ± 7.0	24.2 ± 3.2			Not to change their previous lifestyle			
Kim et al., 2015 [37]	Exp	0/18	74.4 ± 2.9	25.3 ± 2.2	12	2 (100%)	elastic band training (2–3 s × 12–15 r)	BORG 12–13	Yes (V/I)	Serum ELISA ^c^ (48 h)
	Con	0/14	76.0 ± 5.7	23.8 ± 3.0			Not to change their lifestyle and perform stretching exercises once a week for one hour			
Zhao et al., 2017 [38]	Exp	10/0	62.3 ± 3.5	NS	12	3 (100%)	6 machine-based exercises (2–4 s × 5–12 r) + 5 CORE exercises	70–85% 1RM	Yes (I)	Serum ELISA ^d^ (NS)
	Con	7/0	61.9 ± 3.1	NS			No exercise intervention			
Ghanbari-Niaki et al., 2018 [39]	Exp	0/12	58.0 ± 4.7	26.6 ± 3.1	8	3 (100%)	12 exercise circuit training (2 s × 30″)	55% 1RM	No	Serum ELISA ^e^ (48 h)
	Con	0/12	56.5 ± 4.2	27.9 ± 2.2			Not to change their usual care			
Korkmaz et al., 2019 [40]	Exp	36/0	54 ± 6.1	30.3 ± 3.2	12	3 (100%)	8 machine-based exercises + 6 bodyweight exercises	85% 1RM	Yes (I)	Plasma ELISA ^c^ (NS)
	Con	40/0	54 ± 7.2	28.6 ± 3.0			Not to change their lifestyle			
Amanat et al., 2020 [41]	Exp	0/14	54.5 ± 6.9	29.0 ± 2.9	12	3 (100%)	10 machine-based exercises (2 s × 8–10 r)	75–80% 1RM	Yes (V/I)	Serum ELISA ^c^ (24 h)
	Con	0/14	54.5 ± 6.9	29.1 ± 4.6			Not to change their habitual physical activity			
Dianatinasab et al., 2020 [42]	Exp	0/13	53.5 ± 6.5	29.5 ± 2.9	8	3 (100%)	10 machine-based exercises (2 s × 8–10 r)	75–80% 1RM	Yes (V/I)	Serum ELISA ^c^ (24 h)
	Con	0/15	53.5 ± 6.5	30.4 ± 3.8			Not to change their habitual physical activity			

1RM: one repetition maximum; BMI: Body Max Index; Con: control group; D: intervention duration; Exp: experimental group; ELISA: Enzyme-linked immunosorbent assay; F: exercise frequency (per wk.); I: exercise intensity; m/f: male/female; NS: not stated; P: progression; V: exercise volume. ^a^ Age and BMI, data are expressed as means ± standard deviations. ^b^ Represents the time periods for taking blood samples after the last training session. ^c^ The ELISA kit used was obtained from Phoenix Pharmaceuticals Inc., Burlingame, CA, USA. ^d^ The ELISA kit used was obtained from BioVendor-Laboratorni Medicine Inc., Karasek, Crech Republic. ^e^ The ELISA kit used was obtained from CUSABIO Technology LLC., Houston, TX, USA.

**Table 2:** Summary assessments of the risk of bias for each study according to the revised Cochrane risk-of-bias tool for randomized trials, RoB 2.

Study, Year	Randomization Process	Deviations from Intended Interventions	Missing Outcome Data	Measurement of the Outcome	Selection of the Reported Result	Overall Judgment
Hecksteden et al., 2013 [35]	LR	LR	LR	LR	LR	**LR**
Scharhag-Rosenberger et al., 2014 [36]	LR	LR	LR	SC	LR	**SC**
Kim et al., 2015 [37]	LR	LR	LR	SC	LR	**SC**
Zhao et al., 2017 [38]	LR	LR	LR	SC	LR	**SC**
Ghanbari-Niaki et al., 2018 [39]	LR	LR	LR	SC	LR	**SC**
Korkmaz et al., 2019 [40]	LR	LR	LR	SC	LR	**SC**
Amanat et al., 2020 [41]	LR	LR	LR	LR	LR	**LR**
Dianatinasab et al., 2020 [42]	LR	LR	LR	LR	LR	**LR**

LR: low risk of bias; SC: some concerns; HR: high risk of bias. Overall judgment, LR: low risk of bias for all domains; SC: some concerns in at least one domain; HR: high risk of bias in at least one domain or some concerns for multiple domains. The overall judgement for each article is highlighted in bold type in the last column.

**Table 3:** Influence of moderator variables in the chronic effect of resistance training on circulating irisin.

Variable	Subgroup	Groups	*n*	Effect Size with 95% Confidence Interval	Test for Subgroup Effect ^a^	Test for Subgroup Differences ^b^
Age	Adults	5	230	0.04 [−0.64, 0.73]	*p* = 0.91, I^2^ = 83.0%	*p* < 0.001 I^2^ = 93.6%
	Older adults	2	49	2.01 [1.31, 2.70]	*p* < 0.001, I^2^ = 0.0%	
Gender	Male	1	17	1.98 [0.81, 3.15]	*p* < 0.001 I^2^ = NA	*p* = 0.17 I^2^ = 47.3%
	Female	4	112	0.96 [0.11, 1.82]	*p* = 0.03, I^2^ = 78.0%	
Change in body weight (kg)	Increase	0	0	Not estimable	Not applicable	*p* = 0.36 I^2^ = 0.0%
	Same	3	131	0.40 [−1.13, 1.93]	*p* = 0.61, I^2^ = 93.0%	
	Decrease	3	69	1.20 [0.44, 1.95]	*p* = 0.002, I^2^ = 51.0%	
Change in BMI (kg/m^2^)	Increase	0	0	Not estimable	Not applicable	*p* = 0.93 I^2^ = 0.0%
	Same	2	60	1.00 [−0.97, 2.97]	*p* = 0.32, I^2^ = 92.0%	
	Decrease	2	52	0.91 [0.23, 1.59]	*p* = 0.009, I^2^ = 28.0%	
Change in body fat (%)	Increase	0	0	Not estimable	Not applicable	*p* < 0.001 I^2^ = 92.3%
	Same	2	99	−0.42 [−1.13, 0.30]	*p* = 0.25, I^2^ = 63.0%	
	Decrease	4	101	1.42 [0.72, 2.12]	*p* < 0.001, I^2^ = 59.0%	
Intervention duration (weeks)	≤12 weeks	5	129	1.13 [0.35, 1.91]	*p* = 0.005, I^2^ = 76.0%	*p* < 0.001 I^2^ = 94.3%
	>12 weeks	2	150	−0.68 [−1.01, −0.35]	*p* < 0.001, I^2^ = 0.0%	
Training intensity	≤60%	3	174	−0.11 [−1.07, 0.86]	*p* = 0.83, I^2^ = 88.0%	*p* = 0.09 I^2^ = 65.9%
	61 to 85%	4	105	1.10 [0.11, 2.09]	*p* = 0.03, I^2^ = 81.0%	
	>85%	0	0	Not estimable	Not applicable	
Progression in intensity	No	3	174	−0.11 [−1.07, 0.86]	*p* = 0.83, I^2^ = 88.0%	*p* = 0.09 I^2^ = 65.9%
	Yes	4	105	1.10 [0.11, 2.09]	*p* = 0.03, I^2^ = 81.0%	
Supervised sessions (%)	≤80%	1	71	−0.73 [−1.21, −0.25]	*p* = 0.003, I^2^ = NA	*p* = 0.004 I^2^ = 88.0%
	>80%	6	208	0.82 [−0.11, 1.76]	*p* = 0.09, I^2^ = 89.0%	
Risk of bias	Low risk	3	135	−0.06 [−0.81, 0.69]	*p* = 0.88, I^2^ = 76.0%	*p* = 0.19 I^2^ = 42%
	Some concerns	4	144	1.10 [−0.46, 2.67]	*p* = 0.17, I^2^ = 93.0%	
Journal rank	Q1	5	227	0.57 [−0.49, 1.63]	*p* = 0.29, I^2^ = 92.0%	*p* = 0.09 I^2^ = 58.5%
	Q2	1	24	1.30 [0.42, 2.18]	*p* = 0.004, I^2^ = NA	
	Q3	0	0	Not estimable	Not applicable	
	Q4	1	28	0.01 [−0.73, 0.75]	*p* = 0.98, I^2^ = NA	

^a^ Two-tailed null hypothesis test (*p* value), Higgins’s I^2^; ^b^ Cochran’s Q test for heterogeneity (*p* value), Higgins’s I^2^; NA: not applicable.

## Data Availability

The dataset generated and analyzed during the Systematic review and Meta-analysis are available on request from the corresponding author.

## Supplementary Materials

The following are available online at https://www.mdpi.com/1660-4601/18/5/2476/s1, Table S1: PRISMA checklist; Figure S1: Search strategies.
