# Supplementary material for: Effect of Chronic Resistance Training on Circulating Irisin: Systematic Review and Meta-Analysis of Randomized Controlled Trials
Source: Int J Environ Res Public Health. 2021 Mar 3;18(5):2476. doi: 10.3390/ijerph18052476 (PMC7967601; doi:10.3390/ijerph18052476)
Supplement: Supplementary file 1 [file ijerph-18-02476-s001.pdf]

Online Supplementary Material

Publisher: MDPI
Journal: International Journal of Environmental Research and Public Health

Effect of chronic resistance training on circulating irisin: Systematic review and meta-analysis of Randomized Controlled Trials

Pedro L. Cosio[1,2*], Manuel Crespo-Posadas[1], Álvaro Velarde-Sotres[1] and Mireia Pelaez[1]

[1] Faculty of Health Sciences, Universidad Europea del Atlántico, 39011 Santander, Spain; pedroluiscosio@gmail.com (P.L.C.); manuel.crespo@uneatlantico.es (M.C.-P.); alvaro.velarde@uneatlantico.es (A.V.-S.); mireia.pelaez@uneatlantico.es (M.P.)
[2] National Institute of Physical Education and Sport of Catalonia (INEFC), University of Barcelona, 08038 Barcelona, Spain
* Correspondence: pedroluiscosio@gmail.com (P.L.C.)

Corresponding author
Pedro L. Cosio
National Institute of Physical Education and Sport of Catalonia (INEFC)
Av. de l'Estadi, 12-22
08038 Barcelona, Spain
pedroluiscosio@gmail.com

Online Supplementary Table S1: PRISMA Checklist

Section/topic	#	Checklist item	Reported on page #
**TITLE**			
Title	1	Identify the report as a systematic review, meta-analysis, or both.	1
**ABSTRACT**			
Structured summary	2	Provide a structured summary including, as applicable: background; objectives; data sources; study eligibility criteria, participants, and interventions; study appraisal and synthesis methods; results; limitations; conclusions and implications of key findings; systematic review registration number.	1
**INTRODUCTION**			
Rationale	3	Describe the rationale for the review in the context of what is already known.	1, 2
Objectives	4	Provide an explicit statement of questions being addressed with reference to participants, interventions, comparisons, outcomes, and study design (PICOS).	2
**METHODS**			
Protocol and registration	5	Indicate if a review protocol exists, if and where it can be accessed (e.g., Web address), and, if available, provide registration information including registration number.	2
Eligibility criteria	6	Specify study characteristics (e.g., PICOS, length of follow-up) and report characteristics (e.g., years considered, language, publication status) used as criteria for eligibility, giving rationale.	2
Information sources	7	Describe all information sources (e.g., databases with dates of coverage, contact with study authors to identify additional studies) in the search and date last searched.	2
Search	8	Present full electronic search strategy for at least one database, including any limits used, such that it could be repeated.	2
Study selection	9	State the process for selecting studies (i.e., screening, eligibility, included in systematic review, and, if applicable, included in the meta-analysis).	3
Data collection process	10	Describe method of data extraction from reports (e.g., piloted forms, independently, in duplicate) and any processes for obtaining and confirming data from investigators.	3
Data items	11	List and define all variables for which data were sought (e.g., PICOS, funding sources) and any assumptions and simplifications made.	3
Risk of bias in individual studies	12	Describe methods used for assessing risk of bias of individual studies (including specification of whether this was done at the study or outcome level), and how this information is to be used in any data synthesis.	3
Summary measures	13	State the principal summary measures (e.g., risk ratio, difference in means).	3
Synthesis of results	14	Describe the methods of handling data and combining results of studies, if done, including measures of consistency (e.g., I^2) for each meta-analysis.	3, 4

Section/topic	#	Checklist item	Reported on page #
Risk of bias across studies	15	Specify any assessment of risk of bias that may affect the cumulative evidence (e.g., publication bias, selective reporting within studies).	4
Additional analyses	16	Describe methods of additional analyses (e.g., sensitivity or subgroup analyses, meta-regression), if done, indicating which were pre-specified.	3, 4
**RESULTS**			
Study selection	17	Give numbers of studies screened, assessed for eligibility, and included in the review, with reasons for exclusions at each stage, ideally with a flow diagram.	4 and Fig. 1
Study characteristics	18	For each study, present characteristics for which data were extracted (e.g., study size, PICOS, follow-up period) and provide the citations.	4, 5, 6 and Table 1
Risk of bias within studies	19	Present data on risk of bias of each study and, if available, any outcome level assessment (see item 12).	6 and Table 2
Results of individual studies	20	For all outcomes considered (benefits or harms), present, for each study: (a) simple summary data for each intervention group (b) effect estimates and confidence intervals, ideally with a forest plot.	7 and Fig. 2
Synthesis of results	21	Present results of each meta-analysis done, including confidence intervals and measures of consistency.	7 and Fig. 2-6
Risk of bias across studies	22	Present results of any assessment of risk of bias across studies (see Item 15).	11
Additional analysis	23	Give results of additional analyses, if done (e.g., sensitivity or subgroup analyses, meta-regression [see Item 16]).	7-10, Fig. 3-6 and Table 3
**DISCUSSION**			
Summary of evidence	24	Summarize the main findings including the strength of evidence for each main outcome; consider their relevance to key groups (e.g., healthcare providers, users, and policy makers).	11-13
Limitations	25	Discuss limitations at study and outcome level (e.g., risk of bias), and at review-level (e.g., incomplete retrieval of identified research, reporting bias).	13
Conclusions	26	Provide a general interpretation of the results in the context of other evidence, and implications for future research.	13
**FUNDING**			
Funding	27	Describe sources of funding for the systematic review and other support (e.g., supply of data); role of funders for the systematic review.	14

From: Moher D, Liberati A, Tetzlaff J, Altman DG, The PRISMA Group (2009). Preferred Reporting Items for Systematic Reviews and Meta-Analyses: The PRISMA Statement. PLoS Med 6(6): e1000097. doi:10.1371/journal.pmed1000097

For more information, visit: **www.prisma-statement.org**.

Online Supplementary Figure S1: Search strategy

Search strategy in PubMed

1 irisin [All fields]
Filter: Publication date from 01/01/2012 to 31/12/2020
2 FNDC5 [All fields]
Filter: Publication date from 01/01/2012 to 31/12/2020
3 exercise [All fields]
Filter: Publication date from 01/01/2012 to 31/12/2020
4 training [All fields]
Filter: Publication date from 01/01/2012 to 31/12/2020
5 resistance training [All fields]
Filter: Publication date from 01/01/2012 to 31/12/2020
6 # 1 AND # 3
7 # 1 AND # 4
8 # 1 AND # 5
9 # 2 AND # 3
10 # 2 AND # 4
11 # 2 AND # 5
12 # 6 OR # 7 OR # 8 OR # 9 OR # 10 OR # 11

Search strategy in EBSCO (Academic Search Complete)

1 irisin [TX All Text]
Filter: Publication date from 01/01/2012 to 31/12/2020
2 FNDC5 [TX All Text]
Filter: Publication date from 01/01/2012 to 31/12/2020
3 exercise [TX All Text]
Filter: Publication date from 01/01/2012 to 31/12/2020
4 training [TX All Text]
Filter: Publication date from 01/01/2012 to 31/12/2020
5 resistance training [TX All Text]
Filter: Publication date from 01/01/2012 to 31/12/2020
6 # 1 AND # 3
7 # 1 AND # 4
8 # 1 AND # 5
9 # 2 AND # 3
10 # 2 AND # 4
11 # 2 AND # 5
12 # 6 OR # 7 OR # 8 OR # 9 OR # 10 OR # 11

Search strategy in Web of Science

1 irisin [All fields]
Filter: Publication date from 01/01/2012 to 31/12/2020
2 FNDC5 [All fields]
Filter: Publication date from 01/01/2012 to 31/12/2020
3 exercise [All fields]
Filter: Publication date from 01/01/2012 to 31/12/2020
4 training [All fields]
Filter: Publication date from 01/01/2012 to 31/12/2020
5 resistance training [All fields]
Filter: Publication date from 01/01/2012 to 31/12/2020
6 # 1 AND # 3
7 # 1 AND # 4
8 # 1 AND # 5
9 # 2 AND # 3
10 # 2 AND # 4
11 # 2 AND # 5
12 # 6 OR # 7 OR # 8 OR # 9 OR # 10 OR # 11
